# The p.R92W variant of *NR5A1/Nr5a1* induces testicular development of 46,XX gonads in humans, but not in mice: phenotypic comparison of human patients and mutation-induced mice

**DOI:** 10.1186/s13293-016-0114-6

**Published:** 2016-11-08

**Authors:** Mami Miyado, Masafumi Inui, Maki Igarashi, Yuko Katoh-Fukui, Kei Takasawa, Akiko Hakoda, Junko Kanno, Kenichi Kashimada, Kenji Miyado, Moe Tamano, Tsutomu Ogata, Shuji Takada, Maki Fukami

**Affiliations:** 1 Department of Molecular Endocrinology, National Research Institute for Child Health and Development, Tokyo, 157-8535 Japan; 2 Department of Systems BioMedicine, National Research Institute for Child Health and Development, Tokyo, 157-8535 Japan; 3 Department of Reproductive Biology, National Research Institute for Child Health and Development, Tokyo, 157-8535 Japan; 4 Department of Pediatrics and Developmental Biology, Tokyo Medical and Dental University (TMDU), Tokyo, 113-8510 Japan; 5 Department of Endocrinology, Miyagi Children’s Hospital, Sendai, 989-3126 Japan; 6 Department of Pediatrics, Hamamatsu University School of Medicine, Hamamatsu, 431-3192 Japan

**Keywords:** Disorders of sex development, Genome editing, Gonadal development, Gonadal dysgenesis, Mouse model, Mutation, Sex differentiation, SF-1

## Abstract

NR5A1 is the key regulator of adrenal and gonadal development in both humans and mice. Recently, a missense substitution in human *NR5A1*, p.R92W, was shown to underlie gonadal dysgenesis in genetic males and testicular formation in genetic females. Here, we investigated the phenotypic effects of the p.R92W mutation on murine development. Mice carrying the p.R92W mutation manifested a similar but milder phenotype than that of the previously described *Nr5a1* knockout mice. Importantly, mutation-positive XX mice showed no signs of masculinization. These results, together with prior observations, indicate that the p.R92W mutation in *NR5A1/Nr5a1* encodes unique molecules that disrupt male gonadal development in both humans and mice and induces testicular formation specifically in human females. Our findings provide novel insights into the conservation and divergence in the molecular networks underlying mammalian sexual development.

## Introduction

Nuclear receptor subfamily 5 group A member 1 (NR5A1) plays a critical role in the development of the adrenal gland and gonad in human [1, 2]. Heterozygous loss-of-function mutations in *NR5A1* account for a certain percentage of the etiology of gonadal dysgenesis in 46,XY individuals and a small fraction of the genetic causes of ovarian insufficiency in 46,XX individuals [1–3]. *NR5A1* mutations rarely underlie adrenal insufficiency, indicating that during human development, gonads are more vulnerable than adrenal glands to the reduced NR5A1 activity [1, 2]. Recently, Bashamboo et al. identified a heterozygous missense mutation in *NR5A1*, p.R92W (c.274C > T), in four unrelated patients with 46,XX testicular/ovotesticular disorders of sex development (DSD) [4]. This mutation affects a highly conserved amino acid in the DNA binding domain of the wild-type (WT) NR5A1 protein. These findings provide the first indication that specific *NR5A1* mutations can switch the developmental processes of immature 46,XX gonads toward testicular formation. Since Bashamboo et al. identified p.R92W in two phenotypically normal mothers of patients and a 46,XY sibling with female-type external genitalia [4], this mutation seems to be associated with a broad phenotypic spectrum. Subsequently, the same mutation was identified in additional 46,XX DSD patients [5, 6].

Murine NR5A1 has 94 % amino acid homology with human NR5A1 and is involved in adrenal and gonadal development [1, 2, 7, 8]. Homozygous *Nr5a1* knockout (*Nr5a1* ^−/−^) mice lack the adrenal gland and gonad and die within 8 days after birth [7, 8]. Heterozygous knockout (*Nr5a1* ^WT/−^) mice are viable and show no apparent abnormalities, except for mildly compromised glucocorticoid secretion and relatively small testes and ovaries [8, 9]. These observations imply that NR5A1 plays similar roles in humans and mice, although there are some inter-species differences in the functions of this protein in various tissues. Thus, it remains unknown whether the p.R92W mutation affects sexual development in mice.

Genome-editing is a new technology that enables researchers to introduce specific nucleotide substitutions into the genome [10]. This technology is useful to create animal models of human disorders. In our previous study, we performed genome-editing to generate mice carrying the p.R92W mutation of *Nr5a1* [10]. Here, we analyzed phenotypic characteristics of homozygous (*Nr5a1* ^p.R92W/p.R92W^) and heterozygous (*Nr5a1* ^WT/p.R92W^) mice with this mutation.

## Materials and methods

### Animal treatment

This study was approved by the Animal Care Committee at the National Research Institute for Child Health and Development (project number: A2016-002). All experiments were performed in accordance with the institutional guidelines of the care and use of laboratory animals.

### Generation of mice carrying the p.R92W substitution

Prior to this study, we introduced the p.R92W mutation into the murine genome using the CRISPR/Cas9 system [10]. In brief, guide RNAs, hCas9 mRNA, and single-stranded donor oligonucleotides were injected into BDF1 zygotes. The cytosine at the 274th position was successfully substituted to a thymine. Mice were genotyped by PCR sequencing using genomic DNA samples extracted from tail tips.

### Phenotypic analysis of mice carrying the p.R92W substitution

Mutation-positive mice of two separate lines were backcrossed to the C57BL/6N strain for at least five generations. Mice of the fifth or sixth generations were analyzed in this study. Their age-matched WT littermates were used as controls. We examined external genitalia of homozygous (*Nr5a1* ^p.R92W/p.R92W^, *n* = 100) and heterozygous (*Nr5a1* ^WT/p.R92W^, *n* = 100) fetal and neonatal mice. Since homozygosity of the p.R92W mutation resulted in early postnatal death, we performed cesarean section at 18.5 days post-coitum (dpc) to obtain tissue samples from *Nr5a1* ^p.R92W/p.R92W^ mice. Urogenital organs and adrenal glands of the fetuses were subjected to morphological analyses, and gonads were analyzed histologically (46,XX, *n* = 3; 46,XY, *n* = 3). We also histologically examined gonads obtained from fetuses shortly after sex determination (at 13.5 dpc; 46,XX, *n* = 3; 46,XY, *n* = 3).

In addition, we obtained adrenal glands and gonads from *Nr5a1* ^WT/p.R92W^ mice at 8 weeks of age. These tissues were weighed and subjected to morphological analyses. Statistical differences in the mean values between the two groups were examined by Student’s *t* test or Mann-Whitney’s *U* test. *P* values of less than 0.05 were considered significant. Subsequently, the tissues were sectioned, stained with hematoxylin-eosin, and analyzed histologically.

## Results

### Phenotypic analysis of mice carrying the p.R92W substitution

**Fig. 1:**
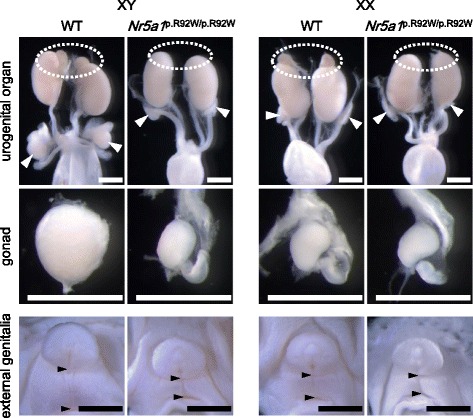
Morphological findings of wild-type (WT) and *Nr5a1* ^p.R92W/p.R92W^ mice. Urogenital organs obtained from XY and XX mouse fetuses at 18.5 days post-coitum. *Broken circles* and *white arrowhead* indicate the positions of adrenal glands and gonads, respectively. *Black arrowheads* depict the ano-genital distance. *Scale bars*, 1 mm

**Fig. 2:**
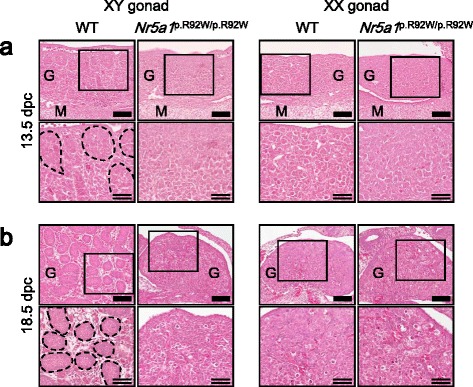
Histological findings of wild-type (WT) and *Nr5a1* ^p.R92W/p.R92W^ mice. Gonads obtained from XY and XX mouse fetuses at 13.5 days post-coitum (dpc) (**a**) and at 18.5 dpc (**b**). Enlarged images are shown in the lower panels. *Black-dashed lines* indicate the testicular cords. *G* gonad, *M* mesonephros. *Single-stranded scale bars*, 100 μm; *double-stranded scale bars*, 50 μm

Mutation-positive mice from the two lines showed identical phenotypes. *Nr5a1* ^p.R92W/p.R92W^ mice were born alive, but died within the first 1 week after birth. *Nr5a1* ^p.R92W/p.R92W^ mice had female-type external genitalia, regardless of karyotype (Fig. 1). Adrenal glands were absent (Fig. 1). *Nr5a1* ^p.R92W/p.R92W^ XY mice manifested hypoplastic gonads in the intra-abdominal region, whereas *Nr5a1* ^p.R92W/p.R92W^ XX mice had gonads of normal or slightly reduced sizes (Fig. 1). Histological analyses revealed that while the testicular cords were recognizable in the gonads of WT XY mice at 13.5 and 18.5 dpc, such structures were not apparent in the gonads of *Nr5a1* ^p.R92W/p.R92W^ XY mice at both stages (Fig. 2). There were no noticeable differences in gonadal histology between *Nr5a1* ^p.R92W/p.R92W^ and WT XX mice at 13.5 and 18.5 dpc (Fig. 2).

**Fig. 3:**
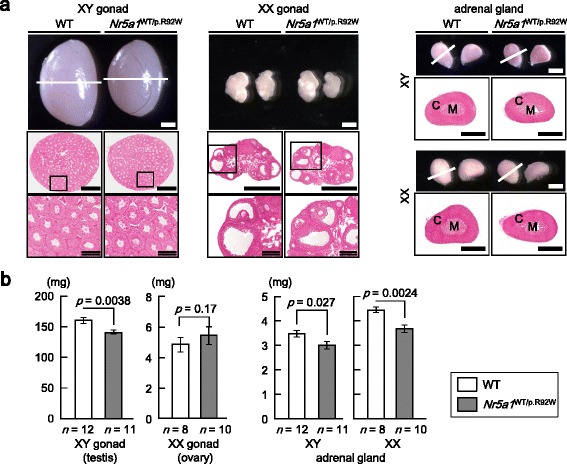
Morphological and histological findings of wild-type (WT) and *Nr5a1* ^WT/p.R92W^ mice. **a** Gonads and adrenal glands obtained from mice at 8 weeks of age. Enlarged images are shown in the *lower panels. C* cortex, *M* medulla. *Single-stranded scale bars*, 1 mm; *double-stranded scale bars*, 200 μm. **b** Weights of gonads and adrenal glands in WT and *Nr5a1* ^WT/p.R92W^ mice at 8 weeks of age. Data are expressed as the mean ± SEM

*Nr5a1* ^WT/p.R92W^ mice were healthy and virtually indistinguishable from the WT littermates. *Nr5a1* ^WT/p.R92W^ mice of both sexes showed karyotype-matched external genitalia and were fertile. However, *Nr5a1* ^WT/p.R92W^ mice at 8 weeks of age showed somewhat smaller adrenal glands than those of WT animals (Fig. 3a, b). Furthermore, testicular size was mildly reduced in *Nr5a1* ^WT/p.R92W^ XY mice, whereas ovarian size was comparable between *Nr5a1* ^WT/p.R92W^ and WT XX mice (Fig. 3a, b). Testes and ovaries of *Nr5a1* ^WT/p.R92W^ mice were histologically unremarkable (Fig. 3a).

## Discussion

We investigated the phenotypic characteristics of mice carrying the p.R92W mutation in *Nr5a1*, an orthologous substitution of a unique human mutation that underlies gonadal dysgenesis in 46,XY patients and testicular formation in 46,XX patients [4–6]. The phenotypes of mutation-positive mice were similar but slightly milder than those of the previously reported *Nr5a1* ^−/−^ mice [1, 2, 7, 8]. Indeed, *Nr5a1* ^p.R92W/p.R92W^ mice exhibited hypoplastic gonads and a lack of adrenal glands. Neonatal death of *Nr5a1* ^p.R92W/p.R92W^ mice can be attributed to glucocorticoid deficiency, as in the case of *Nr5a1* ^−/−^ mice [7, 8]. Likewise, adult *Nr5a1* ^WT/p.R92W^ mice had relatively small adrenal glands and testes compared with those of the WT animals, although they were healthy and fertile. Again, these phenotypes were comparable to those in previously described *Nr5a1* ^WT/−^ mice, which exhibited no obvious abnormalities except for subnormal gonadal size and subnormal cortisol production [8, 9]. On the other hand, our *Nr5a1* ^WT/p.R92W^ and *Nr5a1* ^p.R92W/p.R92W^ XX mice retained apparently normal ovaries, whereas *Nr5a1* ^WT/−^ XX mice are known to have undersized ovaries [9]. It appears that the p.R92W mutation exerts a minor effect on ovarian development.

The present study revealed similarities and differences in the phenotypic effects of the p.R92W mutation in humans and mice. In both species, the p.R92W mutation caused XY gonadal dysgenesis. Defective testicular development in genetic males with this mutation is indicative of the impaired transactivation activity of the mutant NR5A1 on *SOX9*/*Sox9*. Consistent with this, in vitro assays have shown that the p.R92W mutant barely transactivates the testis enhancer sequence core element (TESCO) of *Sox9* [4]. In this regard, the p.R92W mutation appears to abolish the NR5A1 function also in the adrenal gland, as evidenced by the lack of adrenal gland in *Nr5a1* ^p.R92W/p.R92W^ mice. While mutation-positive human patients showed no signs of adrenal insufficiency, the mutation may still impair NR5A1 function in human adrenal glands because *NR5A1* haploinsufficiency usually permits normal adrenal function in humans [1, 2].

In contrast, the p.R92W mutation did not cause apparent XX testicular DSD in mice. Indeed, *Nr5a1* ^p.R92W/p.R92W^ XX fetuses showed histologically unremarkable ovaries at 13.5 and 18.5 dpc. This suggests a functional difference of the p.R92W mutation between humans and mice, although the number of gonadal samples analyzed in the present study was relatively small. Since Bashamboo et al. identified the p.R92W mutation in both 46,XX DSD patients and their unaffected mothers [4], this mutation may have low penetrance. Thus, we examined external genitalia of more than 50 *Nr5a1* ^WT/p.R92W^ XX mice and confirmed that none of these animals had masculinized external genitalia. The discrepancy in ovarian phenotypes between human patients and mutation-induced mice may reflect the inter-species differences in the expression pattern of *NR5A1/Nr5a1* in immature ovaries. *Nr5A1* transcripts are barely detectable in the murine ovary during the critical period for gonadal formation, whereas *NR5A1* mRNA is continuously expressed in the human fetal ovary [4, 11]. It has been suggested that in the human fetal ovary, NR5A1 activates several anti-testis genes, such as *CTNNB* and *WNT4* [5], and antagonizes nuclear receptor subfamily 0 group B member 1 (NR0B1) to suppress *SOX9* expression [4, 6]. NR5A1-induced suppression of *SOX9* appears to be essential for the maintenance of normal ovarian development in humans [4]. It is worth mentioning that simple loss-of-function mutations in human *NR5A1* is insufficient to induce testicular development in genetic females. Indeed, although various nonsense and frameshift mutations in *NR5A1* have been reported to date [1, 2], none of these mutations caused 46,XX testicular DSD. Therefore, the p.R92W protein should have a unique activity that alters the developmental switch of human immature ovary toward testicular formation. It is possible that p.R92W affects the expression levels of anti-testis genes in the developing human ovary [5]. Alternatively, the p.R92W protein may be less sensitive to NR0B1-induced suppression on *SOX9* TESCO compared to WT NR5A1 [6]. These assumptions need to be validated in future studies.

In conclusion, the results of this study, in conjunction with previous observations [4–6], imply that the p.R92W mutation in *NR5A1/Nr5a1* disrupts male gonadal development in both humans and mice and induces aberrant testicular formation specifically in human females. These findings provide novel insights into the conserved and species-specific molecular networks underlying mammalian sex development.
